# Whole-Genome Sequence of a Beijing Extensively Drug-Resistant *Mycobacterium tuberculosis* Clinical Isolate from Buenaventura, Colombia

**DOI:** 10.1128/genomeA.01549-15

**Published:** 2016-01-14

**Authors:** N. Alvarez, D. Haft, U. A. Hurtado, J. Robledo, F. Rouzaud

**Affiliations:** a Corporación para Investigaciones Biológicas (CIB), Medellín, Colombia; b J. Craig Venter Institute, Rockville, Maryland, USA; c Universidad Pontificia Bolivariana (UPB), Medellín, Colombia; d Equal Opportunity Life Sciences (EQUOLS), Rockville, Maryland, USA

## Abstract

Extensively drug-resistant *Mycobacterium tuberculosis* (XDR-TB) has been reported to the WHO by 100 countries, including Colombia. An estimated 9.0% of people with multidrug-resistant TB have XDR-TB. We report the genome sequence of a Beijing XDR-TB clinical isolate from Buenaventura, Colombia. The genome sequence is composed of 4,298,162 bp with 4,359 genes.

## GENOME ANNOUNCEMENT

In 2013, the global tuberculosis (TB) incidence was 126 cases per 100,000 population, corresponding to 9.0 million incident cases worldwide, of which 480,000 were multidrug-resistant TB (MDR-TB) (1). The same year, TB caused about 1.5 million deaths worldwide. By the end of 2012, 92 countries reported extensively drug-resistant TB (XDR-TB) cases to WHO, and 100 countries reported it in 2013. An estimated 9.0% of MDR-TB cases were XDR-TB (1). The continued increase and spread of XDR-TB cases is becoming a serious threat to public health worldwide, as well as to the long-term elimination target set for 2050.

Studies in South America have revealed that the Beijing family accounts for 1% to 2% of the clinical isolates (2, 3). However, occasionally rates as high as 5% or 10% have been reported in Peru and Colombia, the latter being in the harbor city of Buenaventura (4, 5). To better understand the molecular mechanisms involved in the extensive drug resistance of *M. tuberculosis* strains from the Beijing family, we sequenced the whole genome of a sputum clinical isolate from Buenaventura, Colombia.

24-locus MIRU VNTR genotyping was used to confirm the assignment of isolate TBR-103 to the *M. tuberculosis* Beijing lineage (6). Phenotypic susceptibility tests were performed using the Bactec MGIT 960 method. It was determined that TBR-103 is resistant to isoniazid, rifampin, ethambutol, streptomycin, ofloxacin, moxifloxacin, and amikacin, and therefore is classified as XDR-TB. Genomic DNA was obtained from isolate TBR-103 by the CTAB method (7). Samples were treated with RNase prior to paired-end library construction with an average target insert size of 800 bp. Whole-genome sequencing was performed at the J. Craig Venter Institute (JCVI) in Rockville, Maryland, USA, on a MiSeq Illumina platform with 250-bp reads. The genomic DNA was sequenced to about 70× coverage. The draft whole-genome sequence is composed of 4,298,162 bp and 64.94% GC with an *N*_50_ contig length of 60,737 bp. The Celera Assembler was used to perform a *de novo* assembly. JCVI’s in-house pipeline, which uses multiple ranked sources of evidence, including the TIGRFAMs and Pfam protein family databases, provided structural and functional annotation. The assembly contained 4,359 protein-coding genes, along with 45 tRNAs. Mutations associated with resistance to rifampin (S450L in *rpoB*), isoniazid (S315T and R463L in *katG*), ethambutol (M306V in *embB*), and streptomycin (K43R in *rpsL*) were encountered. Also, several SNPs associated with resistance to aminoglycosides were found, such as A1401G in the *rrs* gene, as well as the deletions 805A and 808C in the *eis* gene. No mutation associated with resistance to fluoroquinolones was detected in *gyrA* or *gyrB* genes.

This is the second whole-genome sequencing report of a Beijing XDR-TB isolate circulating in Colombia (8). This will provide a foundation for comparative genomic analysis with XDR-TB genomes circulating in the Americas, as well as in other regions of the world (9–13).

### Nucleotide sequence accession numbers.

This genome project has been deposited at DDBJ/EMBL/GenBank under the accession number JRJT00000000. The version described in this paper is the first version, JRJT01000000.
